# A comparative study of different activation methods for hydrochar: surface properties and removal of pharmaceutical pollutant in water

**DOI:** 10.1007/s11356-025-36706-8

**Published:** 2025-07-11

**Authors:** Arshitha Madhusudhan, Tomas Zelenka, Leonid Satrapinskyy, Tomas Roch, Maros Gregor, Peng Cheng, Olivier Monfort

**Affiliations:** 1 Department of Inorganic Chemistry, Faculty of Natural Sciences, Comenius University Bratislava, Ilkovicova 6, Mlynska Dolina, 842 15 Bratislava, Slovak Republic; 2 Department of Chemistry, Faculty of Sciences, University of Ostrava, 30. dubna 22, 701 03 Ostrava, Czech Republic; 3 Centre for Nanotechnology and Advanced Materials, Faculty of Mathematics, Physics and Informatics, Comenius University Bratislava, Bratislava, Mlynska Dolina 842 48 Slovak Republic; 4 Université Clermont Auvergne, CNRS, Institut de Chimie de Clermont-Ferrand, Clermont-Ferrand, F-63000 France; 5 Department of Environmental Engineering, School of Resources and Environmental Science, Wuhan University, Wuhan, 430079 PR China

**Keywords:** Adsorption, Carbon, Wastewater, Sulfamethoxazole, H_2_O_2_

## Abstract

Contaminants of emerging concerns, such as sulfonamides, have been frequently discovered in surface water, and the design of biomass-based adsorbents is a promising research direction to remove them from water. In this study, hydrothermal carbonization was utilized to prepare hydrochar (HC) from orange peels along with various activations, including hydrogen peroxide (H_2_O_2_) and HCl. The main goals of the present study are (i) to prepare hydrochars that were activated by different chemical methods and (ii) to provide insights into the adsorption mechanism of pollutant removal using sulfamethoxazole (SMX) as a model pharmaceutical pollutant. The H_2_O_2_ activated hydrochar (ACHC) exhibited the best adsorption capacity for removal of 40 μM SMX from water, i.e., 1.971 mg g^−1^ using 0.2 g L^−1^ ACHC. Scanning electron microscopy (SEM) studies revealed that the ACHC exhibited a coral-like structure and the highest amount of mesopores (74.6 m^2^ g^−1^) and BET area of 79.5 m^2^ g^−1^. The effects of pH, adsorbent dosage, initial concentration, and adsorption temperature were investigated, and a substantial relationship between porosity and adsorption suggests that mesoporosity played a crucial role in the adsorption process for all the activated hydrochars. The mechanism of SMX adsorption involves reversible chemisorption and retention in the pores of the adsorbent surface. The use of ACHC was also tested in different water matrices to highlight its potential applications in wastewater treatment, and it exhibited an adsorption capacity of 0.598 and 0.429 mg g^−1^ in tap water and wastewater effluents, respectively.

**Supplementary Information:**

The online version contains supplementary material available at 10.1007/s11356-025-36706-8.

## Introduction

Antibiotics are requisite pharmaceuticals used to prevent and cure infections from bacteria in humans and animals (Hossain et al. 2022). However, most ingested antibiotics are not entirely metabolized, and a large part can be transferred into the natural environment, especially surface waters due to not efficient wastewater treatment plants (WWTPs) (Le et al. 2023). Sulfamethoxazole (SMX) is a bacteriostatic antibiotic derivative of sulfonamides that belongs to the most widely detected antibiotic in wastewater, with a half-life of 85–100 days (Rana et al. 2023) and improvement of treatment technologies to remove SMX and other pharmaceuticals is required to achieve the limits fixed by policymakers like the European Commission (Directive 2024/3019 of the European Parliament and Council concerning Urban Wastewater Treatment).

Among the possible treatments, several studies have been focused on biological treatments of pharmaceuticals in wastewaters (Zahedi et al. 2022). However, antibiotics such as SMX can reduce long-term survival of bacteria used in such treatments and thus negatively impact their overall efficiency. Consequently, the used bacteria can generate an excess of sludge that must be removed from the system to maintain a suitable efficiency (Adeoye et al. 2024). In addition, biological treatments operate over a long time compared to other types of treatment (Crini and Lichtfouse, 2019). Chemical techniques, such as advanced oxidation processes (AOPs), can operate in an aqueous phase and break down efficiently organic pollutants using highly reactive species like hydroxyl radicals. These short-lifetime species destroy the target pollutants into intermediate products but sometimes they can be even more toxic. Furthermore, due to the presence of organic matter in wastewaters, the reactive species are scavenged, resulting in reduced efficiency (Crini and Lichtfouse, 2019). On the other hand, the technique of adsorption offers advantages over the procedures mentioned above due to its ease of operation, low cost, and low energy consumption (Crini and Lichtfouse, 2019). Carbonaceous compounds such as activated carbon, biochar, and carbon nanotubes have been already investigated for their ability to adsorb various contaminants. Due to these reasons, carbon-based materials are considered one of the most effective treatments.

To this end, the design of biomass-based adsorbents is a potential research direction to explore. Biomass is a lignocellulosic material made from living organic resources such as wood and fruits/vegetables. The classification of biomass feedstock (wet or dry) for the manufacture of char is critical for the choice of pre-treatment technology. Vegetable and fruit wastes, sewage sludge, animal wastes, algae, and so on, have a high moisture content (> 30%) and are thus referred to as “wet biomass,” whereas agricultural residues and a few wood species have a low moisture content (< 30%) and are thus classified as “dry biomass.” There are two major techniques to convert biomass into char. Pyrolysis is used to prepare biochar, which is a solid material obtained from the thermochemical conversion of biomass in an oxygen-limited environment according to the International Biochar Initiative (Kambo and Dutta 2015). On the other hand, hydrochar is manufactured using hydrothermal carbonization (HTC) of a slurry containing the biomass. The HTC process, which is employed to prepare biochar, is carried out in the presence of water, so the high moisture content of the biomass feedstock is not a limiting factor. This distinct benefit eliminates the need for wet biomass pre-drying, which is a large energy-consuming procedure and a financial burden in biomass pre-processing. In addition, the HTC process yields 40–70% hydrochar with low ash content in comparison to biochar production. Although hydrochar research is still in its infancy in terms of process optimization, tailored design for specific applications, and understanding of its water treatment potential, it is a promising pathway to support the transition to a circular economy since biomass-based wastes can be reused to treat wastes as in WWTP. However, hydrochar differs from biochar by its low porosity, thus requiring an activation process.

The primary goal of the activation process is to improve the surface area, pore volume, pore diameter, and porosity of the resulting hydrochar. One of the most often used procedures for producing activated hydrochar is chemical activation via acidic and alkaline activation to generate surface functional groups and enhance the hydrophilicity. Acidic activation is often done to increase the surface concentration of oxygenated functional groups while decreasing the point of zero charge (pH_PZC_). Biochar treated with strong acids such as HNO_3_ and H_2_SO_4_ showed a loss in total surface area and porosity due to pore wall rupture and the expansion of micro-pores into meso- or macro-pores during acid erosion (Yakout 2015). In contrast, activation with H_2_O_2_ improved the meso-pore volume of biochar. For instance, Zhang et al. have increased the specific surface (by 15.3%) as well as the oxygen content (16.4%–22.3%) of hydrochar by using hydrogen peroxide (H_2_O_2_) (Zhang et al. 2020). Alkaline activation increases the presence of hydrophilic functional groups and pH_PZC_ (Panwar and Pawar 2022). Although each of these chemical activation methods has its advantages/disadvantages based on the targeted pollutant, a comparative study of different acidic and alkaline routes has not been yet performed and might bring additional insight in the fundamental science related to the understanding of hydrochar along with their potential integration in WWTPs.

The present study focuses on synthesizing hydrochar from orange peels using the HTC process, followed by different chemical activation methods. For comparison of the different activated hydrochars, surface morphology, chemical content, and physical characteristics have been considered. The activated hydrochars have been evaluated as an adsorbent for the removal of SMX in water, and the effect of different experimental parameters, including initial concentration, temperature, pH, catalyst concentration, and contact time, has been assessed. Insights into the adsorption mechanism of the best system have been provided as fundamental understanding work which is a first stage to consider before potential applications.

## Materials and methods

### Chemical and synthesis of hydrochars

Sulfamethoxazole (SMX, C₁₀H₁₁N₃O₃S, ≥ 98.0%) was purchased from Merck. Hydrogen peroxide (H_2_O_2_, 30 wt%) and sodium hydroxide (NaOH) were acquired from CentralChem, Slovakia, while hydrochloric acid (HCl, ACS reagent, 37%) was purchased from Merck. The organic solvents used for HPLC, including methanol (MeOH, HPLC grade) and water (H_2_O, HPLC grade) were bought from Merck. The solutions were prepared using deionized water (DI, 16.0 MΩ cm). Oranges, i.e., the source of carbon for hydrochar preparation, were purchased at a local market.

Orange peels were cut, dried at 80 °C, crushed, milled, and sieved (0.5–1 mm) henceforth referred to as hydrochar precursor (OP). A mass of 3 g of hydrochar precursor was soaked in 30 mL of DI water for 30 min in a Teflon container, and its hydrothermal conversion was performed once it was transferred into a stainless-steel autoclave heated at 160 °C for 12 h under autogenerated pressure conditions. After cooling to ambient temperature, the solid was recovered by centrifugation and washed with distilled water before being dried at 80 °C. The produced hydrochar is referred to as HC henceforth, and three different activated hydrochars were prepared.

Chemical activations (Table 1) of HC were done using either HCl or H_2_O_2_. For HCl modification (AHC), 1 M of HCl was added directly to the solution in which OP was soaked, and then, HTC was performed under the same conditions as above. For H_2_O_2_ modification (ACHC), 1 g of the final HC sample was immersed in a 25 mL 10% H_2_O_2_ solution for 2 h at room temperature. The activated hydrochars were rinsed with DI water and dried at 80 °C. In addition, the activated hydrochars were compared with a biochar (NHC) prepared by thermal activation of ACHC hydrochar in a tube furnace under nitrogen atmospheric conditions for 2 h (600 °C at 5 °C/min).

**Table 1:** Nomenclature of the samples

Adsorbents	Type and method of adsorbent activation
HC	Hydrochar:hydrothermal conversion of orange peels (OP)
ACHC	Chemical activation of HC using H_2_O_2_
AHC	Chemical activation of HC using HCl acid
NHC	Thermal activation of ACHC under nitrogen atmosphere

### Characterization methods

The surface morphology of hydrochars was investigated using scanning electron microscope (SEM, Tescan Lyra III) equipped with energy dispersive X-ray spectroscopy (EDX) to investigate the elemental composition. The phase identification was performed using X-ray diffractometer (XRD) (PANanalytical, Cu Kα, *λ* = 1.5418 Å). Surface functional groups were studied using Fourier transform infrared spectroscopy (FTIR) using Thermo Scientific Nicolet 6700 FTIR spectrometer and X-ray photoelectron spectroscopy (XPS) using Omicron multiprobe system equipped with hemispherical mirror analyzers and monochromatic Al Kα radiation). These analyses were considered to compare the structural differences between the activated hydrochars.

The specific surface area is also a key parameter in surface chemistry. The textural properties of the samples were obtained by the measurement of the N_2_ (− 196 °C) and CO_2_ (0 °C) adsorption–desorption isotherms using a static manometric adsorption instrument (Quantachrome Instruments®) as in Kryeziu et al. (2022). Before the physisorption measurements, samples were outgassed at 130 °C under ultra-high vacuum for 20 h. The volume and area of micropores and mesopores were calculated from the pore size distribution (PSD) curves obtained by fitting nitrogen adsorption isotherms with a hybrid QSDFT (Quenched Solid Density Functional Theory) adsorption kernel assuming slit-shaped micropores and cylindrical mesopores. The N_2_ adsorption isotherms were also used to calculate the BET area (*S*_BET_), respecting Rouquerol’s method (Thommes et al. 2015). The CO_2_ adsorption isotherms were fitted by a carbon GCMC (Grand Canonical Monte Carlo) equilibrium transition kernel assuming slit-shaped micropores to generate PSD curves and calculate the micropore volume and area. Information about these methods can be found in Reference (Zelenka et al. 2023). In addition, the point of zero charge (pH_PZC_) for the samples was determined by the pH drift method where the variation of initial pH from 3 to 11 was adjusted by HCl and NaOH. The final equilibrium pH was determined 48 h after the addition of 10 mg of hydrochar (Fig. S1). These characteristics are important to evaluate electrostatic interactions between the pollutant and the adsorbent in the solution (Crini et al. 2007). The PZC determines the surface charge of the adsorbent material at a given pH, which in turn influences the adsorption behavior of different pollutants using their pKa. In other words, the PZC gives information whether electrostatic interactions can be considered during the adsorption.

### Adsorption of sulfamethoxazole

Adsorption experiments were carried out by introducing 10 mg of adsorbent (HC, ACHC, AHC hydrochars and NHC biochar) to 50 mL of 40 μM SMX solution at natural pH at a controlled temperature of 25 °C for 120 min under air bubbling. To quantify the residual concentration of SMX in aqueous solution, 0.5 mL of solution was collected at regular time intervals, filtered through a 0.45-µm PTFE membrane microfilter, and analyzed using HPLC (High Performance Liquid Chromatography, Hitachi-Merck, AS-2000 autosampler, L-6200A pump and L-4250 UV–VIS detector) equipped with a C18 column (Hypersil Gold—Thermo Fisher Scientific). The mobile phase for HPLC analysis was a mixture of MeOH/H_2_O at *v*/*v* = 50:50 in isocratic mode (flow rate of 1 mL min^−1^). The detection wavelength for SMX was set at 268 nm.

The amount of adsorbed SMX was calculated by Eq. 1 where *q*_*t*_ represents the amount of SMX adsorbed per unit mass of hydrochar (mg g^−1^) at time *t* (min), *C*_0_ and *C*_*t*_ represent the initial concentration of SMX (mg L^−1^) and concentration at time *t*, *m* represents the experimental adsorbent mass (g), and *V* represents the volume of SMX solution (L).

1 $${q}_{t}= \frac{\left({C}_{0}-{C}_{t}\right)V}{m}$$

The effect of initial pH (3.0–11.0), temperature (25, 35, 45, 55 °C), catalyst dosage (0.1–1.0 g L^−1^), and SMX concentration of 4–200 μM has been investigated. The adsorption kinetic studies were conducted using a reaction mixture of 50 mL SMX solution and varied doses of hydrochar. The effect of contact time (from 0 to 120 min) was also investigated using different SMX concentrations (4, 20, 40, 80, and 200 μM). The above experiments were performed in triplicates. The mechanism of adsorption was proposed based on FTIR spectroscopy and XPS before and after adsorption to investigate the chemical changes at the surface of the hydrochar. The experimental adsorption kinetics curves were primarily fitted by Crank’s intraparticle diffusion model) (Zelenka, 2016) to calculate the effective diffusion parameter *D*_*e*_ (min^−1^):

2 $$\frac{{q}_{t}}{{q}_{e}}=1-\left(\frac{6}{{\pi }^{2}}\right)\sum\nolimits_{n=1}^{10}\left(\frac{1}{{n}^{2}}\right)\text{exp}\left(-{D}_{e}{n}^{2}{\pi }^{2}t\right)$$

where *q*_*t*_ and *q*_*e*_ are the amount of SMX (mg g^−1^) adsorbed at time *t* (min) and equilibrium, respectively. The term *q*_*t*_*/q*_*e*_ is normalized sorption progress describing the fractional attainment of equilibrium at each step. The *n* is the number of terms of the series; the range *n* = 1–10 was used. The effective diffusion parameter *D*_*e*_ is occasionally referred to as effective diffusivity (Zelenka, 2016).

Experimental adsorption data were also fitted by pseudo-first-order model (PFO) (Simonin 2016):

3 $$\frac{{q}_{t}}{{q}_{e}}=1-{\text{exp}}^{-{k}_{1}t}$$

where* k*_*1*_ is the pseudo-first order rate constant (min^−1^) with the same unit as *D*_*e*_. The advantage of this form (*q*_*t*_*/q*_*e*_) is having a single unknown parameter (*k*_*1*_) (Revellame et al. 2020), so that Eqs. (2) and (3) have only one unknown parameter (*D*_*e*_*, k*_1_), ensuring a fairer model comparison. *D*_*e*_ and *k*_1_ were calculated by non-linear regression using Solver in MS Excel 2016. Akaike information criterion (AIC) (Parzen et al. 1998) and the coefficient of determination (*R*^2^) were evaluated to assess the suitability of the models.

To provide further insights into the adsorption mechanism, desorption of SMX from the hydrochars was performed by adjusting the pH to alkaline conditions (pH = 10) after a 120-min adsorption step. In addition, we have tested the best system in effluents collected at municipal WWTP in Bratislava with defined physico-chemical characteristics (Table S1).

## Results and discussion

### Structural and textural properties of hydrochars

**Fig. 1:**
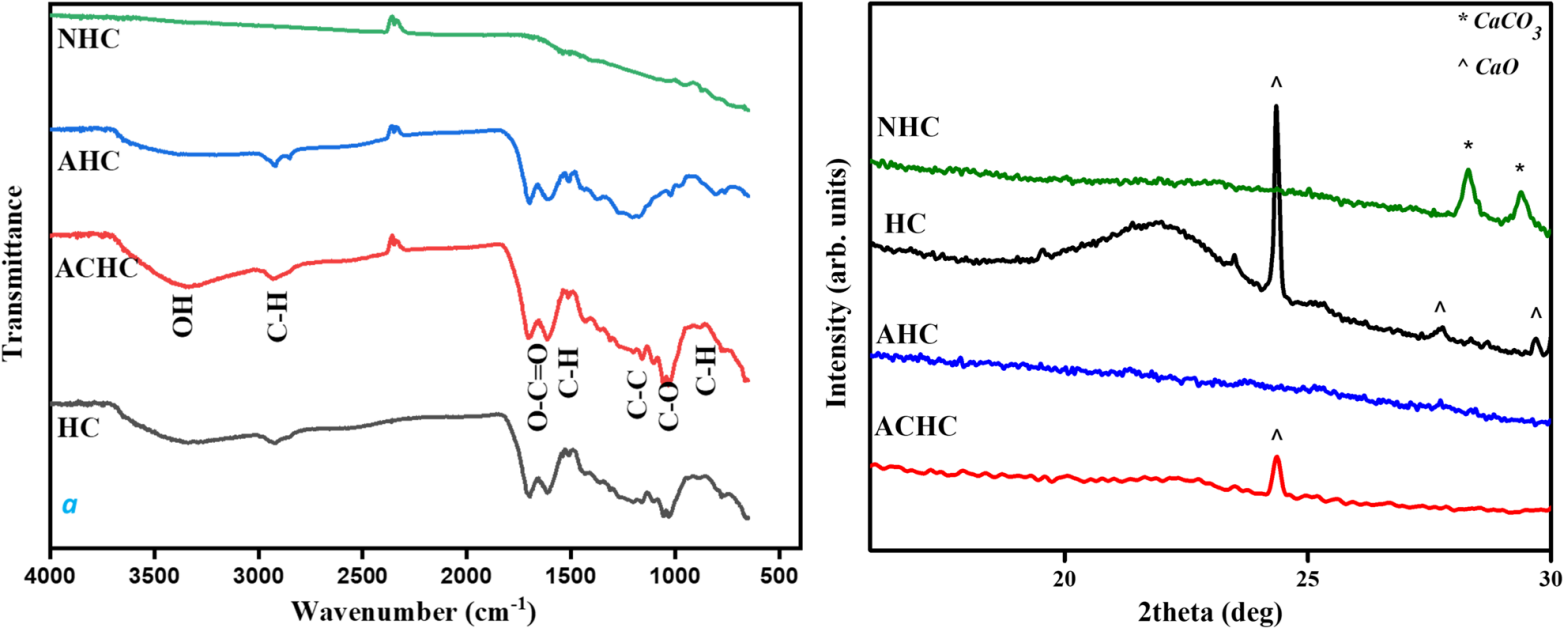
FTIR spectra (left) and XRD patterns (right) of HC, ACHC, AHC, and NHC

The surface modifications upon activation of HTC for the derived ACHC, AHC hydrochars were studied using an FTIR spectrum and compared to pristine hydrochar (HC) and biochar (NHC) (Fig. 1). Except for the NHC sample, the ACHC, AHC, and non-activated HC exhibit the same surface functional groups. The broad and high intensity of spectra at 3340–3440 cm^−1^ indicate O–H stretching vibrations resulting from lower carbonization during the HTC process (Melo et al. 2017). Two brief peaks at 2923 and 2853 cm^−1^ indicate stretching vibrations of aliphatic C-H in carbonyl, thus implying hydrochars possess aliphatic structures that might be created by aromatization, which is performed by the dehydrogenation of existing cyclic molecules (Zhao et al. 2013). The two peaks at 1710 and 1625 cm^−1^ correspond to C = O stretching of carbonyl and C = C vibrations respectively, which indicates the existence of aromatic rings generated by the dehydrogenation and aromatization during the HTC process (M. Li et al. 2011). The 1518 cm^−1^ peak indicates vibrations of C-H bending during the carbonization process (M. Li et al. 2011), and the peak at 1290 cm^−1^ corresponds to C-O stretching modes resulting from oxygenated functional group transformation during HTC (Melo et al. 2017). The out-of-plane C-H bending vibrations at 865–750 cm^−1^ indicate aromatization (Gao et al. 2013). The FTIR data highlight that the obtained hydrochars are rich in carbon, comprising aromatic structures and aliphatic chains forming functional groups which are due to the dehydration and deoxygenation of the biomass. These functional groups could be identified as carbonyl (R–C = O), carboxyl (-O-C = O), hydroxyl (-O–H), ethers (C–O–C), aromatic, and alkyl groups. Compared to non-activated HC, the ACHC exhibits an enhancement in the intensity of the O–H functional groups whereas the AHC shows the opposite, i.e., a decrease in this function. On the other hand, the NHC sample does not contain the majority of the observed functional groups in HC, ACHC, and AHC due to pyrolysis that decomposes these functional moieties.

The XRD patterns of pristine HC, activated hydrochars (ACHC, AHC), and biochar (NHC) exhibit a broad diffraction in the 20–25° 2*θ* range (Fig. 1). This broad diffraction in HC greatly diminished upon activation due to a lower degree of graphitization (Liu and Guo 2015). The width of diffraction maxima indicates a small size of crystalline coherent domains in the order of nanometers. HC and ACHC had other minor peaks corresponding to CaO, which could be present in the precursor peels, and NHC had minor peaks of CaCO_3_, which is a resultant of CaO formed at high temperatures in an inert atmosphere. Therefore, the XRD data confirmed no graphitic structures, and hence an amorphous form of carbon has been produced after HTC and activation methods.

To confirm the high carbon content of the activated hydrochars, EDX analysis has been performed (Table 2) and compared with the non-activated hydrochar and its precursor (orange peels—OP). The HTC process of biomass eliminates a fraction of the oxygen from the feedstock by decarboxylation and dehydration processes, resulting in a decrease in oxygen quantity from OP to HC. Carbon content increased further following the chemical activation phase, and especially after the heat treatment in nitrogen (pyrolysis) in which the majority of oxygen is stripped (Table 2). This observation on NHC sample is confirmed by the FTIR spectroscopy data where almost no functional groups are present.

**Table 2:** Atomic weigth percentage (wt%) of adsorbents from EDX analysis

Sample	C	O
Orange peels	61.53	38.47
HC	78.10	21.90
ACHC	83.86	16.14
AHC	87.04	12.96
NHC	96.83	3.17

**Fig. 2:**
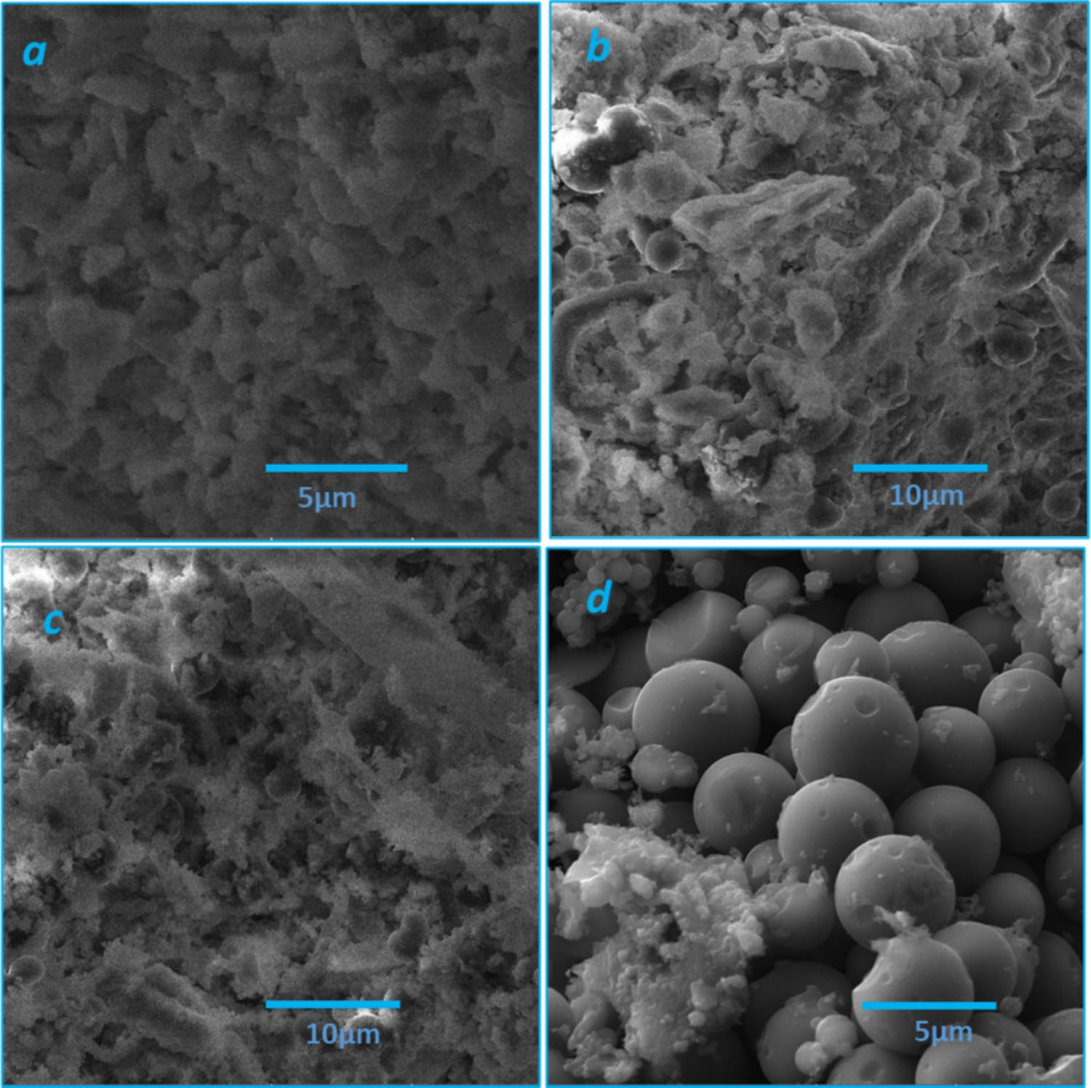
SEM images of **a** HC, **b** ACHC, **c** AHC, and **d** NHC

The surface morphology of the hydrochars is depicted in Fig. 2. The non-activated hydrochar (HC) has an aggregated structure with sizes in the range of a few microns (Fig. 2a). Such a structure with microparticles of various sizes and shapes is related to the HTC process due to the disintegration of cellulosic components and recombination of cellulose fragments (Fernandez et al. 2015). After the chemical activation, ACHC and AHC produce coral-like structures with aggregate sizes comparable to the HC sample (Fig. 2b, c). In other words, the fused microparticles in HC have formed a hierarchical structure of larger microspheres, thus forming a coral-like structure. Upon pyrolysis of activated hydrochar (NHC), the surface morphology significantly changed (Fig. 2d) with the formation of spherical particles of about 5 µm in size. The NHC sample also contains tiny irregular deformations (Fig. 2d).

**Fig. 3:**
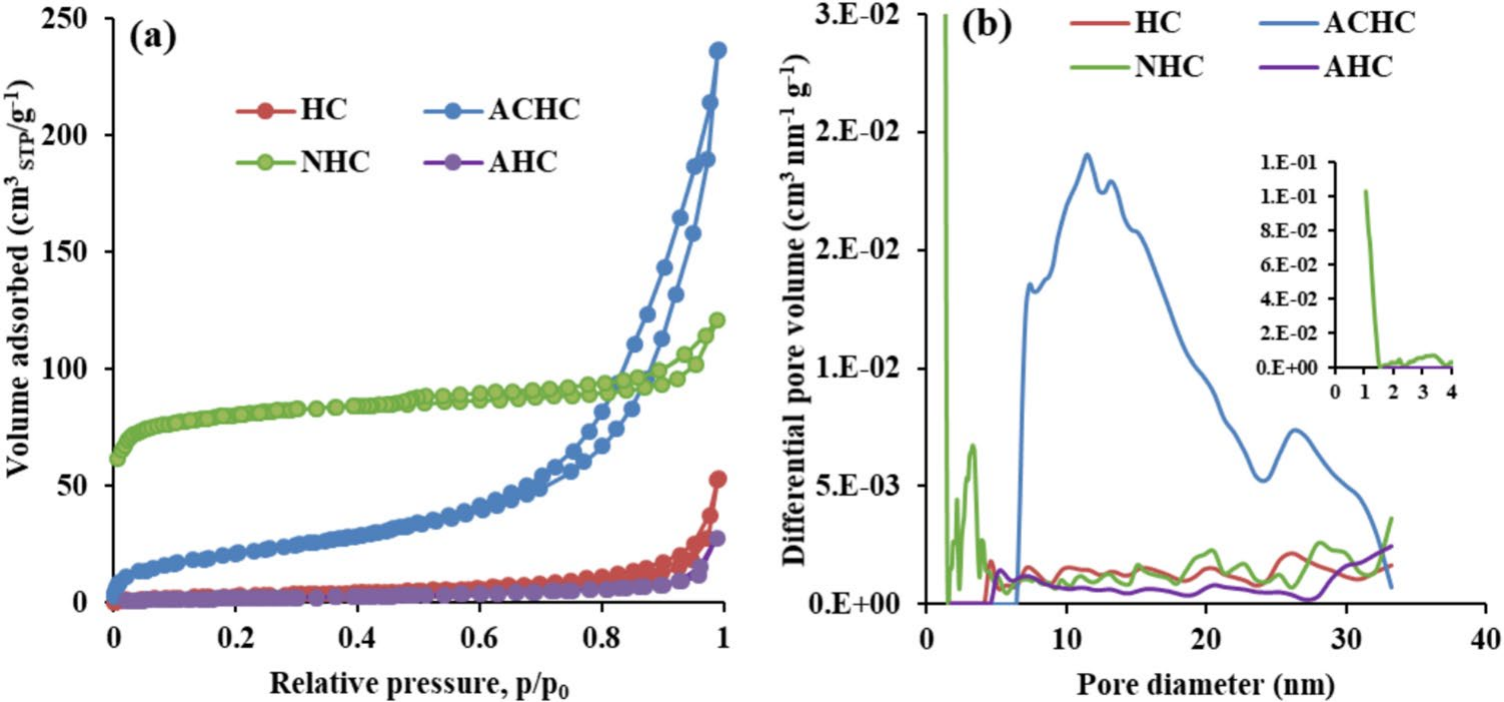
**a** N_2_/− 196 °C adsorption–desorption isotherms and **b** corresponding pore size distributions by QSDFT adsorption kernel. The inset shows a magnified view of pores smaller than 4 nm

**Fig. 4:**
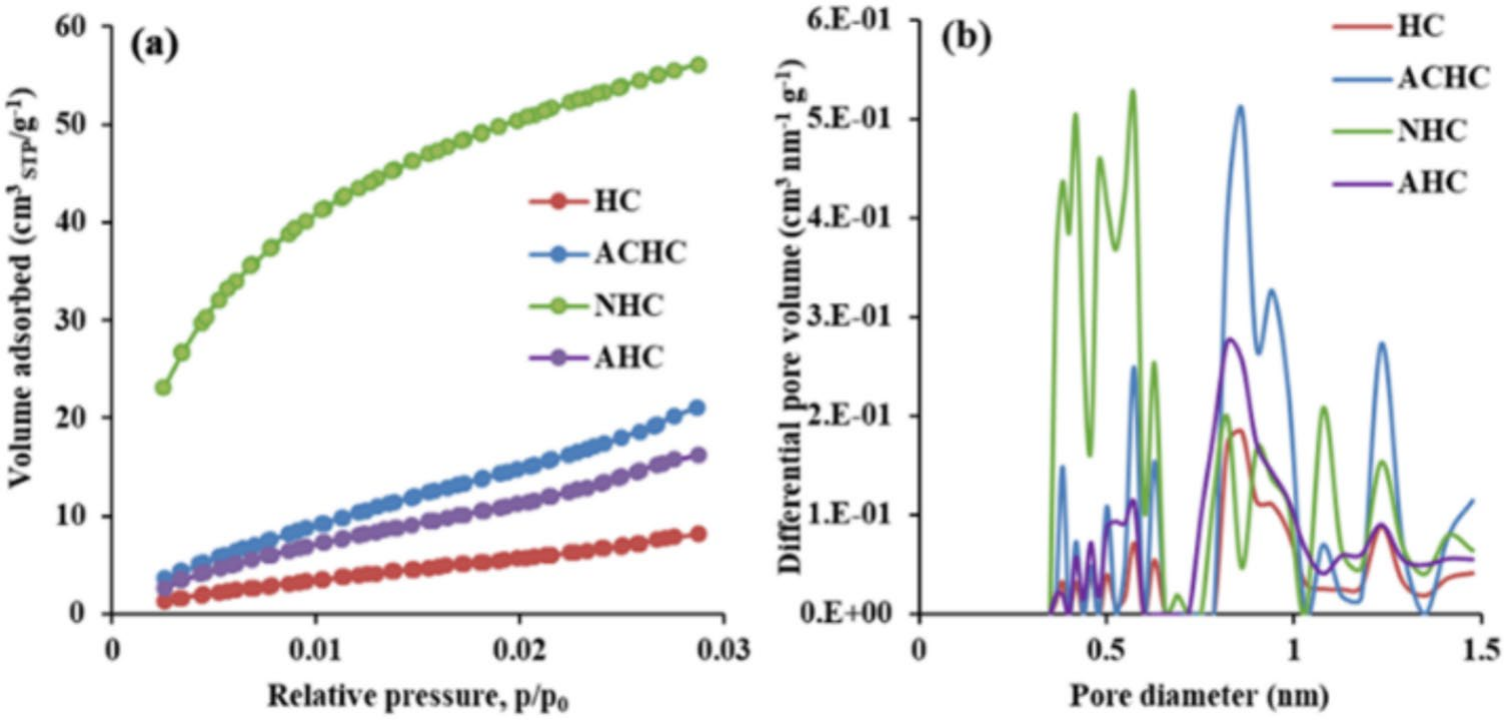
**a** CO_2_/0 °C adsorption–desorption isotherms and **b** corresponding pore size distributions by GCMC kernel

Concerning the textural properties of the hydrochars, gas physisorption was first used to evaluate the porous properties of the original HC sample and its activated derivatives (ACHC, AHC) and biochar (NHC). The N_2_/− 196 °C adsorption and desorption isotherms are shown in Fig. 3a, with corresponding pore size distributions in Fig. 3b. The CO_2_ adsorption–desorption isotherms at 0 °C are shown in Fig. 4a, and the respective pore size distributions in Fig. 4b. The calculated textural properties are summarized in Table 3.

**Table 3:** Summary of the textural properties of the samples. Microporosity was determined using both N₂ physisorption at − 196 °C and CO₂ physisorption at 0 °C, while mesoporosity and BET surface area were evaluated through N₂ physisorption at − 196 °C

Adsorbent	Micropore volume by N_2_/CO_2_ (cm^3^ g^−1^)	Micropore area by N_2_/CO_2_ (m^2^ g^−1^)	Mesopore volume (cm^3^g^−1^)	Mesopore area (m^2^ g^−1^)	BET area (m^2^ g^−1^)
HC	0.000/0.051	0.0/114.6	0.038	10.2	12.2
ACHC	0.000/0.123	0.0/284.8	0.269	76.4	79.5
NHC	0.108/0.174	272.6/577.8	0.049	20.6	313.8
AHC	0.000/0.090	0.0/210.7	0.024	6.5	6.9

The non-activated HC sample exhibits a type IVa N_2_ isotherm according to the latest IUPAC report (Thommes et al. 2015), with a mild hysteresis loop, indicating the presence of some mesopores. The minimal adsorption at low relative pressures suggests a lack of nitrogen-accessible micropores at cryogenic temperatures, which aligns with the micropore characteristics summarized in Table 3. Therefore, CO_2_ physisorption measurements at elevated temperature (0 °C) were performed (Wedler and Span 2021) revealing microporosity with a volume of 0.051 cm^3^ g^−1^. The HCl-activated hydrochar (AHC) nearly doubled the micropore volume (as determined by CO_2_ sorption) but reduced the mesopore volume by one-third and the BET area by half compared to the HC sample (Table 3). In contrast, the H_2_O_2_-activated sample (ACHC) exhibited a sevenfold increase in mesoporosity and BET area (both determined by N_2_ physisorption). The pore size distribution of ACHC revealed a broad range of mesopores between 6 and 33 nm (Fig. 3b), and it is possible that the sample contains even larger mesopores (the analysis is limited by the kernel to 33 nm). The H_2_O_2_ activation method substantially enhances mesoporosity. In addition, CO_2_ sorption results show a 2.5-fold increase in microporosity across the entire micropore range compared to the HC sample (Fig. 4b, Table 3). Upon pyrolysis (NHC), the micropore volume increased from 0 to 0.108 cm^3^ g^−1^ (N_2_ sorption) and from 0.051 to 0.174 cm^3^ g^−1^ (CO_2_ sorption), i.e., almost a 3.5-fold increase. This improvement in microporosity of NHC is attributed to the pyrolysis process carried out at 600 °C, which generates micropores (Kryeziu et al. 2022), especially those about 0.5 nm in size (Fig. 4b). While the mesoporosity of the NHC sample increased slightly over the HC sample, it decreased significantly compared to the ACHC precursor, with mesopore volume reduced by more than five times and mesopore area nearly four times smaller. In summary, the preparation of biochar by pyrolysis led to a significant increase in microporosity but a reduction in mesoporosity.

### Factors influencing the sulfamethoxazole removal efficiency

This subsection examines the influence of textural properties, adsorbent dosage, initial SMX concentration, the pH, and the temperature of the systems on the removal efficiency of SMX using HC, ACHC, AHC, and NHC adsorbents. The mechanism of SMX removal is described in the next section.

**Fig. 5:**
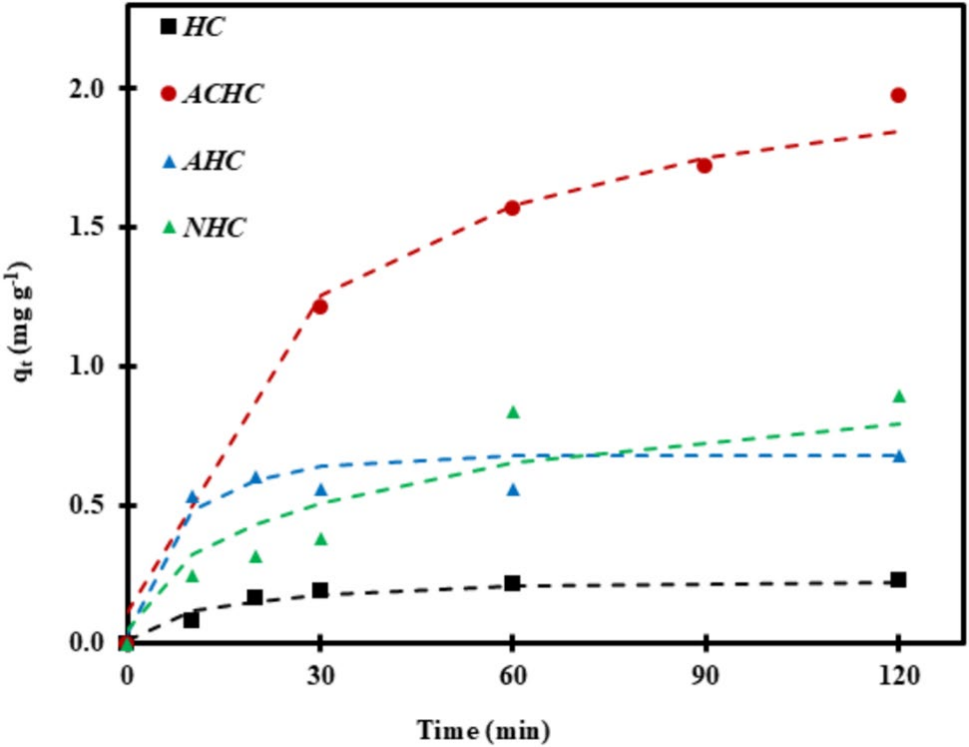
Kinetics of SMX removal using pristine HC, its activated variants (ACHC, AHC), and biochar (NHC). Conditions: [SMX]_0_ = 40 µM, [adsorbent]dosage = 0.2 g L^−1^, *T* = 25 °C. Dashed lines represent fit of Crank’s intraparticle diffusion model

The adsorption kinetics experiments illustrated in Fig. 5 were carried out at natural pH and room temperature (25 °C), with an SMX concentration of 40 μM on both activated and non-activated hydrochars. The adsorption capacity in 120 min was shown to be proportional to the mesopore area, with larger mesopore areas leading to greater adsorption. This large surface area is critical for providing more active sites for SMX adsorption, resulting in an experimental adsorption value of 1.971 mg g^−1^ in ACHC, which has a large mesopore area (76.4 m^2^ g^−1^). A similar pattern was observed even for NHC, which has twice the mesopore area of HC. In contrast, AHC has a reduced mesopore area upon activation while increasing the micropore area, resulting in slightly higher adsorption compared to HC (Fig. 5). Microporosity may also contribute to SMX adsorption, as the micropores could accommodate SMX molecules (width and height < 0.65 nm) (Bizi 2020). However, the majority of micropores in the NHC sample are smaller than 0.6 nm (Fig. 4b) and are therefore unlikely to be accessible for SMX molecules. Additionally, it remains uncertain whether the full extent of microporosity is accessible within the 120 min adsorption period. Summing up, the two activated hydrochars, i.e., ACHC, AHC, and the NHC biochar, exhibited significantly better SMX adsorption than pristine HC due to the formation of micropores and mesopores, resulting in more active adsorption sites.

**Fig. 6:**
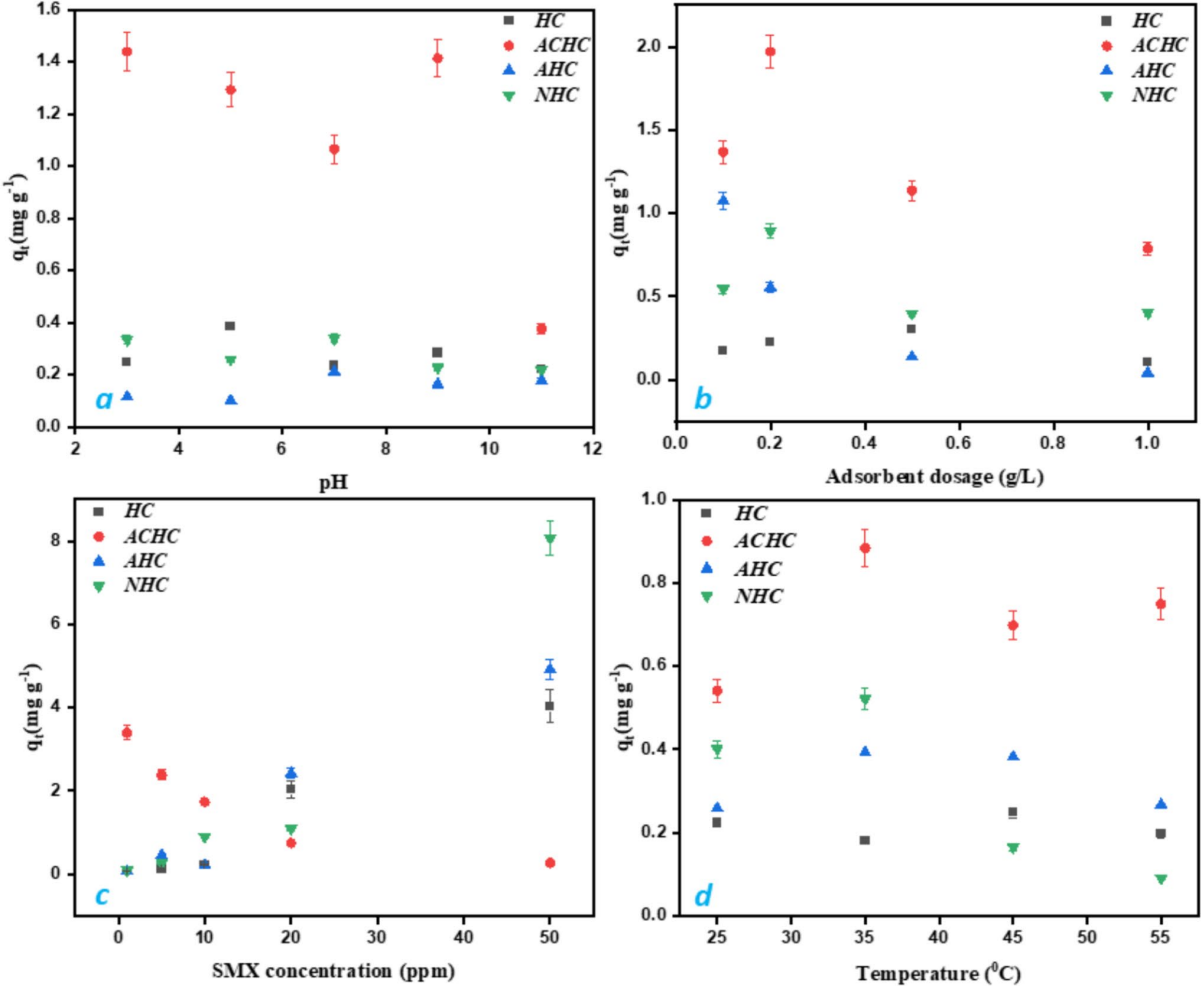
Effect of **a** initial pH (conditions: [SMX]_0_ = 40 µM, [adsorbent]dosage = 0.2 g L^−1^, *T* = 25 °C), **b** adsorbent dosage (conditions: [SMX]_0_ = 40 µM, pH = 7, *T* = 25 °C), **c** SMX concentration ([adsorbent]dosage = 0.2 g L^−1^, pH = 7, *T* = 25 °C), and **d** temperature (conditions: [SMX]_0_ = 40 µM, [adsorbent]dosage = 0.2 g L^−1^, pH = 7) on the adsorption of SMX

The removal of SMX was also studied by varying different experimental conditions (adsorbent dosage, SMX concentration, adsorption temperature, and pH of the system) to assess their impact on the adsorption and to provide insights into the adsorption kinetics of SMX onto differently activated hydrochars (Fig. 6). First, variation of initial pH was investigated (Fig. 6a). The adsorption of SMX was relatively stable whatever the initial pH values, especially for HC, AHC, and NHC. In the case of ACHC, a decrease of SMX adsorption was observed between pH 3 and 7, followed by an increase until pH 10. Thus, for ACHC sample, optimal adsorption efficiency was achieved at pH 3 or 9. This observation suggests different mechanisms are involved based on the pH as discussed in the next section.

The effect of adsorbent dosage was subsequently investigated on the adsorption efficiency of SMX (Fig. 6b). The experiments were carried out at natural pH and room temperature (25 °C) at an SMX concentration of 40 μM. The highest removal of SMX was observed at 0.2 g L^−1^ of ACHC and NHC with a q_t_ value of 1.971 mg g^−1^ and 0.893 mg g^−1^, respectively. In the case of AHC, a continuous decrease of SMX efficiency was observed as the adsorbent dosage increases; thus, the maximum *q*_*t*_ = 1.074 mg g^−1^ was obtained at 0.1 g L^−1^ of AHC. The trend of reduced adsorption capacity at high adsorbent dosage can possibly be due to the presence of additional sites that would support particle aggregation, thus exposing limited active sites for effective SMX adsorption (Shi et al. 2019). The optimal adsorbent dosage of 0.1 and 0.2 g L⁻^1^ can be economically beneficial as it reduces the overall cost of adsorbent material required for efficient SMX removal.

By varying the SMX initial concentration from 4 to 200 µM using 0.2 g L^−1^ of AHC and NHC (Fig. 6c), the adsorption capacities increase, thus highlighting these adsorbents can be also efficient at high SMX concentration given the increased driving forces for mass transport and more adsorption sites. However, the opposite trend was observed for ACHC, thus suggesting such an activated hydrochar is suitable for trace amounts of SMX. Higher concentrations of pollutants could saturate adsorption sites, resulting in reduced capacity of adsorption, which demonstrates a complex interaction between pollutant concentration and adsorption efficiency (Ma et al. 2022).

Temperature-dependent experiments of SMX adsorption were performed at four temperatures: 25, 35, 45, and 55 °C using 40 μM SMX solution and 0.2 g L^−1^ adsorbent at natural pH (Fig. 6d). Results demonstrate that at higher temperatures, the adsorption quantity was decreased due to a decrease in surface activity. The ideal temperature to obtain maximum sulfamethoxazole (SMX) adsorption on activated hydrochars is 35 °C. Multiple studies reveal that the optimal temperature for SMX adsorption is around 35 °C, where the process is spontaneous and exhibits good removal efficiency (Ghate et al. 2021). At temperatures higher than 35 up to 55 °C, the adsorption process becomes exothermic, showing a reduction in adsorption capability as temperature increases (Shi et al. 2019).

To compare the efficiency of ACHC with other C-based adsorbents, CNT, AC, and biochar, Table S2 provided the main characteristics of the removal of SMX and related compounds using different adsorbents. Briefly, the efficiency of our adsorbent appears quite low but is comparable with many of these adsorbents. It is worth noting that the progress in the CNT, AC, and biochar research is more advanced than hydrochar. Consequently, there are many more works focused on the modification and optimization of CNT, AC, and biochar, thus leading to better efficiencies. Since the topic of hydrochar is relatively new and scarcely investigated, we believe our current study might open the doors to future research in this direction. In addition, several points should be taken into consideration for potential scaling up. For instance, CNT and AC are more expensive to produce compared to biochar and hydrochar. Concerning the production costs, CNTs are evaluated at $100/kg on average, whereas activated carbons and biochars cost $1/kg without additional modifications, which are often necessary for the adsorption of pharmaceuticals (Banerjee et al. 2024; De Volder et al. 2013). For treatment costs, all adsorbents imply similar costs, but regarding efficiency, conventional biochar (Table S2) has eightfold better efficiency than our hydrochar. Therefore, further work on hydrochars is required to reduce costs.

### Adsorption mechanism

To elucidate the mechanism of SMX removal, the adsorption via electrostatic interactions is first considered. Therefore, it is crucial to determine the speciation of SMX under the pH conditions of the adsorption experiments, considering its pKa values. The initial pH values were used to assess electrostatic interactions, as the equilibrium pH (measured after 120 min of adsorption) remained unchanged from the initial pH values (ΔpH = 0.3). The SMX molecule has two pKa values (pKa_1_ = 1.7 and pKa_2_ = 5.6) indicating that SMX exists as a cation at pH < 1.7, as an anion at pH > 5.6, and in neutral form between pH of 1.7 and 5.6 (Heo et al. 2019). On the other hand, the point of zero charge (pH_PZC_) of ACHC, AHC, and NHC was determined by the pH drift method (Fig. S1). ACHC has a pH_PZC_ value of 4.46. From Fig. S1, it can be expected that AHC has pH_PZC_ < 3 due to its acidic chemical activation, while pH_PZC_ > 11 for NHC. This increase in PZC upon pyrolysis, which occurs primarily due to a decrease in acidic functional groups, is caused by structural changes in the carbon material as the pyrolysis temperature rises (Sun et al. 2018). By considering the pH_PZC_ of the hydrochars and the pKa values of SMX, it can be stated that physisorption via electrostatic interactions is not the predominant phenomenon (Fig. 6a). This is because the pH range where SMX exists as an anion (pH > 5.6) does not coincide with the range where hydrochars would exhibit positive surface charges (pH < pH_PZC_). Similarly, the neutral form of SMX (pH = 1.7–5.6) and negatively charged hydrochars (pH > pH_PZC_) do not strongly favor electrostatic attractions. This trend does not align with patterns expected from electrostatic interactions. Instead, other mechanisms, such as π-π interactions, hydrogen bonding, or hydrophobic effects, are likely responsible for SMX physisorption onto the hydrochars. For ACHC, at pH 3, adsorption could be predominantly mediated by π-π electron donor–acceptor interactions, while at pH 9, the negative charge-assisted hydrogen bond mechanism could be more prominent. Similar results were found by Li et al. (Li et al. 2022b), though in both cases pore filling is prevalent due to the high surface area and mesoporous structure of the ACHC that facilitates the pore-filling mechanism. In addition, Fig. 6d shows that SMX adsorption increases with temperature up to 35 °C but decreases above that (especially for ACHC, AHC, NHC). Although sorption is generally exothermic and typically decreases with higher temperature (if the process is diffusion-controlled), the adsorption can increase with temperature (Srivastava et al. 2006; Xie et al. 2020). However, above 30 °C, the weakening of adsorbate-adsorbent bonds becomes dominant (Hashemzadeh et al. 2024), which is typical for physical adsorption.

**Fig. 7:**
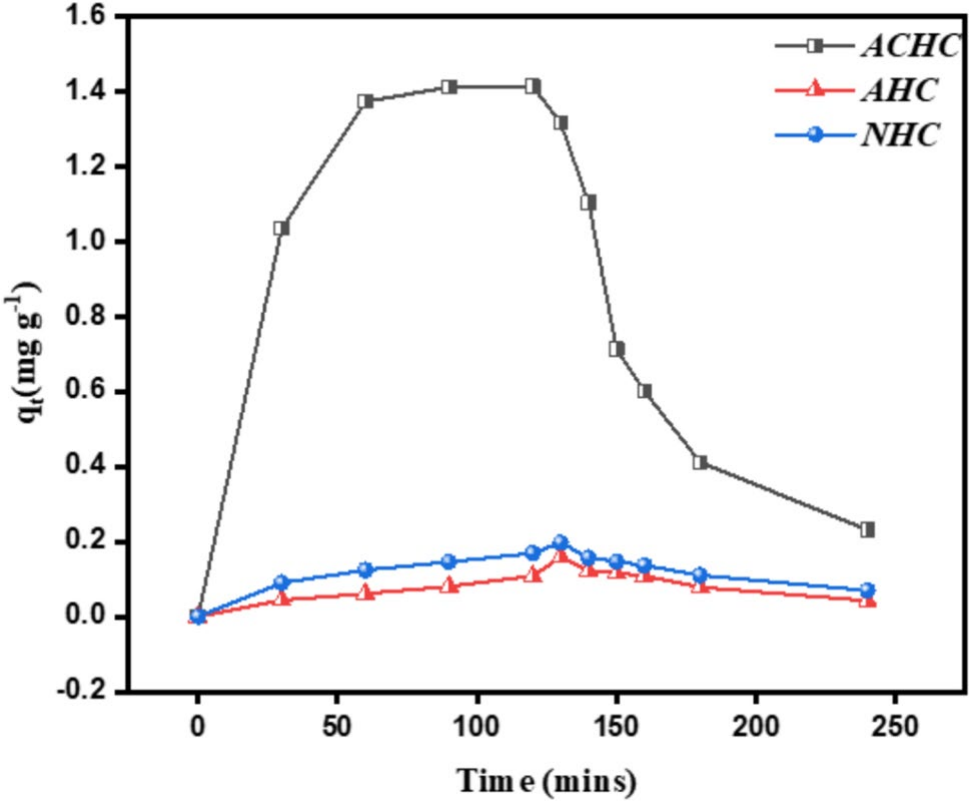
SMX adsorption (0 < *t* < 120 min) and desorption (121 < *t* < 240 min) curves for the activated hydrochars and the biochar (conditions: [SMX]_0_ = 40 µM, [adsorbent]dosage = 0.2 g L.^−1^, *T* = 25 °C, pH = 7)

Results of the SMX desorption experiments may provide another clue on the reversibility of the adsorption process, thus providing further insights into the adsorption mechanism. By inducing SMX desorption at high alkaline pH at *t* = 120 min, the desorption curves (Fig. 7) illustrate the behavior of SMX desorption from three different samples (ACHC, AHC, and NHC). For all three samples, desorption of SMX occurred. After 120 min of desorption, 83%, 61%, and 59% desorption efficiencies were obtained for ACHC, AHC, and NHC, respectively. Therefore, SMX is reversibly bound in the three samples. As discussed above, physisorption by weak interactions and pore filling is probably involved.

To confirm the physisorption and to investigate possible chemisorption, FTIR and XPS analyses of the most promising adsorbent, i.e., ACHC hydrochar, before and after the SMX adsorption were performed to clarify the adsorption mechanism. The FTIR spectrum of ACHC loaded with SMX reveals peak shift and lower intensity (Fig S2). Shifting was seen in the hydroxyl group (-OH) (Zbair et al. 2018) from 3400 to 3340 cm^−1^ proving the formation of hydrogen bond during adsorption, whereas the -COOH peak in 1700 cm^−1^ and -CO peak at 1000 cm^−1^ showed very low intensity in case of ACHC and AHC as proof for π-π attraction (Y. Li et al. 2022a).

**Fig. 8:**
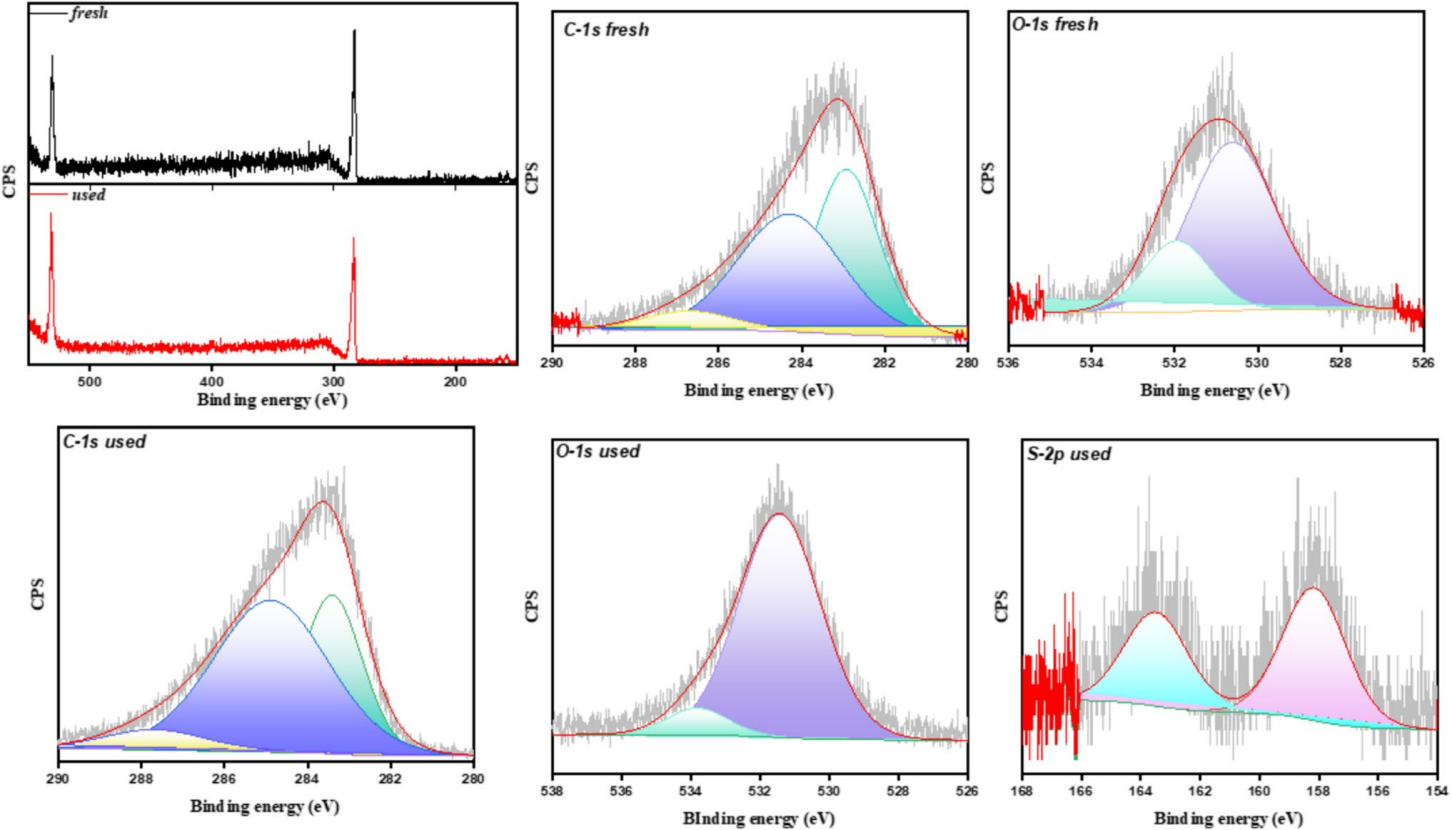
XPS of fresh and used ACHC

**Fig. 9:**
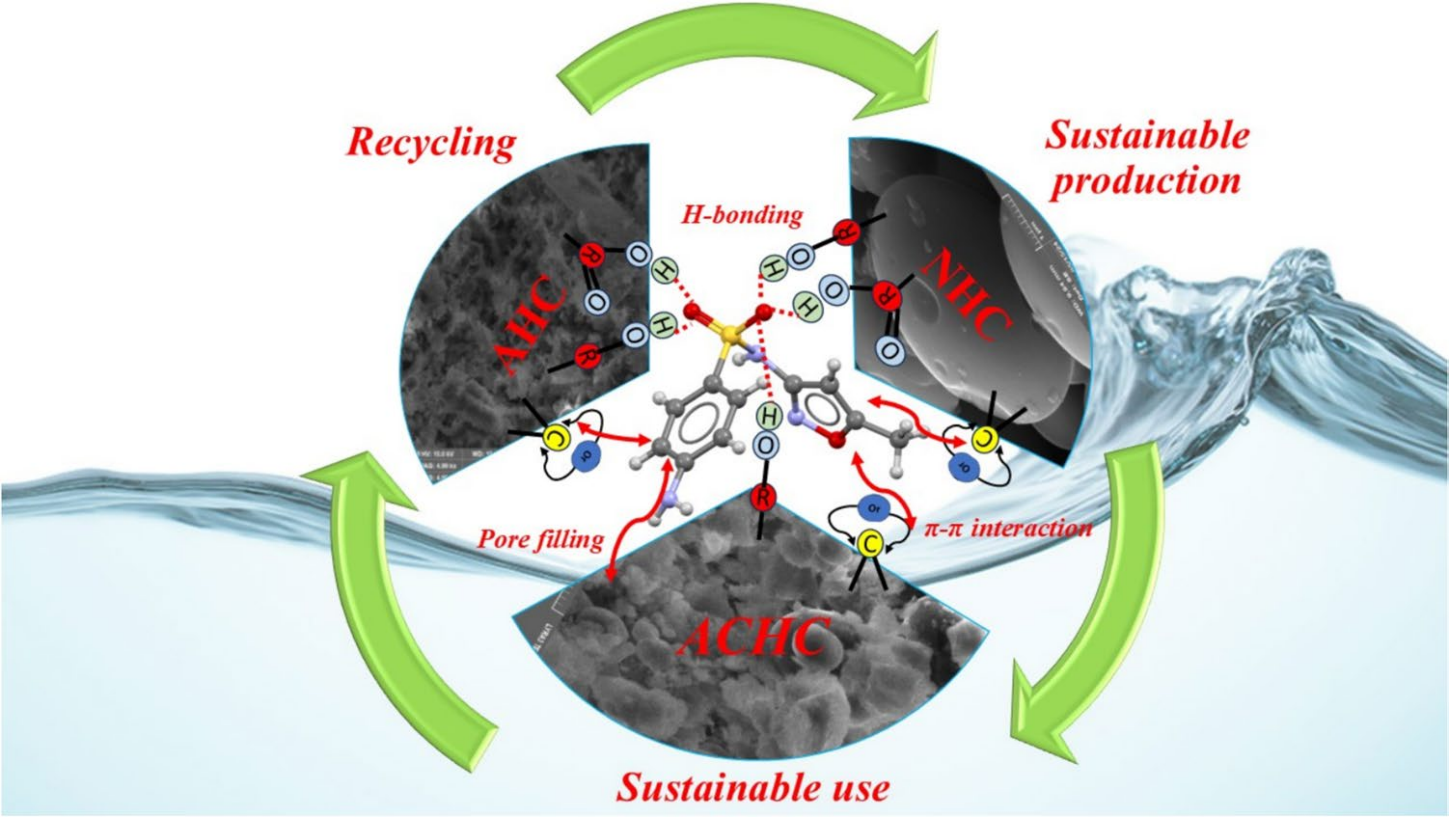
Plausible mechanism for adsorption of SMX on different activated hydrochars

XPS studies were performed on fresh and used (after SMX adsorption) ACHC hydrochar since it is the most promising sample (Fig. 8). The spectrum of the ACHC hydrochar exhibits two peaks: C1s and O1s. In the high-resolution C 1 s spectra, three peaks were observed at 284.83, 286.23, and 288.37 eV, corresponding to the C–C, C-O, and C = O groups, respectively. The high-resolution O 1 s spectra revealed two peaks at 532.19 and 533.85 eV, attributed to the C-O and C = O groups, respectively. Prior to SMX adsorption, no sulfur signal was detected at the surface of ACHC, and an O/C ratio of 0.214 was obtained. After SMX adsorption, the O/C ratio increased to 0.347, and a peak of sulfur appeared, confirming the adsorption of SMX. The results of XPS and FTIR spectroscopy confirmed the physisorption of SMX onto hydrochars, especially ACHC. The full adsorption mechanism is proposed in Fig. 9.

### ACHC hydrochar: adsorption kinetics and tests in wastewater effluents

Adsorption kinetics provides valuable insights into the rate and mechanism of SMX removal. In scientific literature, experimental kinetic data are often fitted using adsorption reaction models such as pseudo-first-order and pseudo-second-order. For physisorption, the mass action is typically a very rapid process and may not significantly impact the kinetic study. Instead, the adsorption process is primarily governed by diffusion mechanisms, such as liquid film diffusion or intraparticle diffusion, with one of these processes acting as the rate-limiting step (as discussed and hypothesized in the previous section). Adsorption diffusion models, such as Crank’s intraparticle diffusion model (Crank 1975), are usually more appropriate as they effectively describe the process of film diffusion and/or intraparticle diffusion of the adsorptive molecule within the adsorbent particles (Qiu et al. 2009). Nevertheless, we chose to nonlinearly fit our data using Crank’s (Eq. 2) and the pseudo-first-order (PFO, Eq. 3) kinetic model for comparison. Both models contain a single unknown parameter each (*D*_*e*_*, k*_*1*_), ensuring a fairer comparison of the models. Additionally, the rate parameters share the same units (min^−1^). The comparison of the fits by Crank’s model and the PFO model is shown in Fig. S3, while the summary of the rate parameters is presented in Table 4. The Akaike information criterion (AIC) and the determination coefficient (*R*^2^) suggest a good fit between the experimental data and the models. The PFO model provides a slightly better fit based on these indicators, though the difference is not significant. Therefore, due to the greater suitability of Crank’s model for describing the adsorption process, it will be preferred for further comparisons. Crank’s model shows the best fit for ACHC hydrochar (*R*^2^ = 0.99, AIC = − 30). The *D*_*e*_ values range from 1.41 × 10^−3^ to 7.61 × 10^−3^ min^−1^ showing insignificant differences. A good fit to Crank’s intraparticle diffusion model and the relatively low diffusion coefficient indicate that SMX transport within ACHC hydrochar probably occurs via intraparticle diffusion.

**Table 4:** The parameters calculated from Crank’s intraparticle diffusion model and the pseudo-first order model (PFO) for the kinetics of SMX removal from Fig. S3. AIC and *R*^2^ refer to Akaike information criterion and determination coefficient, respectively, to evaluate the suitability of a model

Sample	Crank			PFO		
	*D*_*e*_ (min^−1^)	*R*^2^	AIC	*k*_1_ (min^−1^)	*R*^2^	AIC
HC	3.39 × 10^−3^	0.95	− 29	5.67 × 10^−2^	0.99	− 38
ACHC	1.90 × 10^−3^	0.99	− 30	2.86 × 10^−2^	0.99	− 32
AHC	7.61 × 10^−3^	0.91	− 26	1.28 × 10^−1^	0.90	− 25
NHC	1.41 × 10^−3^	0.87	− 22	2.63 × 10^−2^	0.94	− 28
ACHC-DI	9.41 × 10^−3^	0.99	− 41	-	-	-
ACHC-tap water	4.25 × 10^−3^	0.97	− 32	-	-	-
ACHC-wastewater	3.08 × 10^−3^	0.97	− 33	-	-	-

**Fig. 10:**
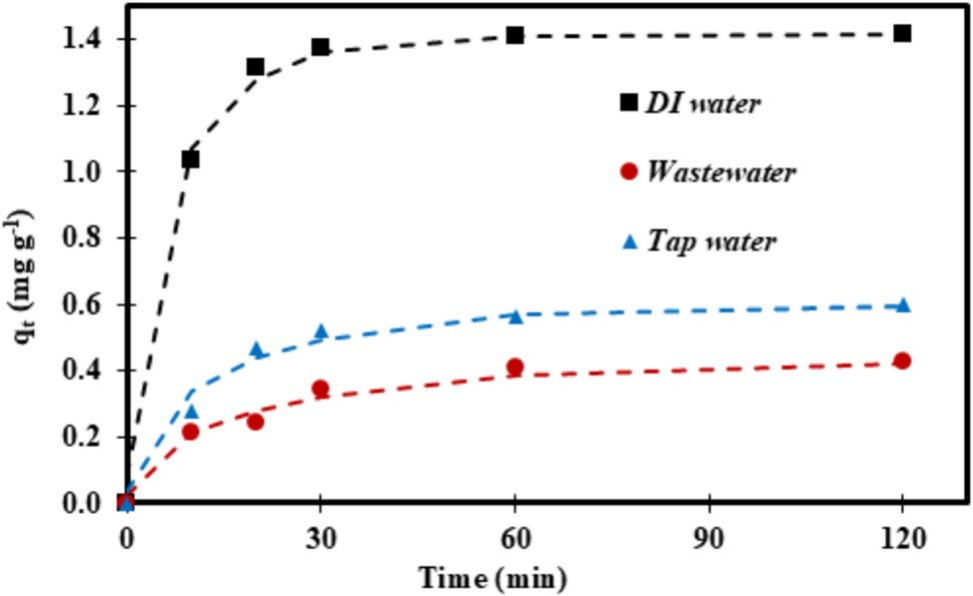
Adsorption kinetics of SMX on ACHC hydrochar in real water matrices. Dashed lines represent the fit of the Crank’s intraparticle diffusion model

To investigate the practical application of the activated hydrochars for adsorption of 40 μM SMX, the performance of 0.2 g L^−1^ ACHC, which was identified as the best adsorbent in the present study, was assessed in both tap water (TW) and effluents from WWTP in Bratislava (WW) as shown in Fig. 10. ACHC demonstrated an effective adsorption performance for SMX in both TW and WW, 0.598 mg g^−1^ and 0.429 mg g^−1^, respectively. These results showed that ACHC could be potentially applied in the treatment of wastewaters. These different water matrices have an expected detrimental effect on SMX adsorption due to its intricate multi-component system. The slightly slower adsorption kinetics of SMX in tap and wastewater compared to DI water (*D*_*e*_ in Table 4) can be attributed to the presence of competing ions and molecules in these water matrices. These substances gradually occupy adsorption sites on the adsorbent surface, reducing the availability of active sites for SMX and thereby slowing down the overall adsorption process.

## Conclusions

For the first time, we provided a comparative study of diverse activations on orange peels. Orange peels were converted employing HTC and the resulting hydrochar was activated chemically with H_2_O_2_ and HCl. For all the activated hydrochars, the adsorption capacity was shown to be related to the mesopore area, with increased mesopore areas resulting in higher adsorption. The use of H_2_O_2_ for activation of hydrochar (ACHC), which was identified as the most efficient activated hydrochar in our study due to its coral shape morphology and the presence of more O–H functional groups on its surface. Adsorption studies showed that, at lower concentrations of 0.2 g L^−1^, aggregation of the hydrochars is limited, thus increasing removal efficiency of the sulfamethoxazole. Also, pore filling has a significant effect on the adsorption process, and it is more effective at lower concentrations as SMX can more easily occupy high-energy sites. This trend was followed for all the three activations. Optimal pH of 3 and 9 were observed for ACHC thus tackling out electrostatic interactions as potential mechanism regarding the pK_a_ of SMX. However, it suggests π-π interactions being more favorable at pH 3 and hydrogen bond interactions at pH 9. At both pH, the pore filling is prevalent due to the high surface and mesoporous area of the ACHC (76.4 m^2^ g^−1^) facilitating such a mechanism. ACHC was tested in different water matrices including tap- and waste-waters. The ACHC hydrochar is considered as a viable and promising adsorbent since an adsorption capacity of 0.429 mg g^−1^ was observed for SMX in effluents of WWTP Bratislava. This study also highlights that energy-intensive pyrolysis, which produces highly microporous materials (NHC), does not necessarily guarantee high adsorption efficiency for SMX adsorption. Finally, this research emphasizes the potential for reusing food waste in wastewater treatment, contributing to the circular economy.

## Supplementary Information

Below is the link to the electronic supplementary material.

ESM 1 DOCX (323 KB)

## Data Availability

The authors declare that the data supporting the findings of this study are available within the paper and its supplementary files. Should any raw data files be needed in another format, they are available from the corresponding author upon reasonable request.
