# Supplementary material for: A comparative study of different activation methods for hydrochar: surface properties and removal of pharmaceutical pollutant in water
Source: Environ Sci Pollut Res Int. 2025 Jul 11;32(30):18107–20. doi: 10.1007/s11356-025-36706-8 (PMC12328538; doi:10.1007/s11356-025-36706-8)
Supplement: Supplementary file 1 — DOCX (323 KB) [file 11356_2025_36706_MOESM1_ESM.docx]

# **A comparative study of different activation methods for hydrochar: surface properties and removal of pharmaceutical pollutant in water**

Arshitha Madhusudhan^a^, Tomas Zelenka^b^, Leonid Satrapinskyy^c^, Tomas Roch^c^, Maros Gregor^c^, Peng Cheng^d,e^, Olivier Monfort^a*^

1. Department of Inorganic Chemistry, Faculty of Natural Sciences, Comenius University Bratislava, Ilkovicova 6, Mlynska Dolina, 842 15 Bratislava, Slovak Republic.
2. Department of Chemistry, Faculty of Sciences, University of Ostrava, 30. dubna 22, 701 03 Ostrava, Czech Republic.
3. Centre for Nanotechnology and Advanced Materials, Faculty of Mathematics, Physics and Informatics, Comenius University Bratislava, Mlynska Dolina, 842 48 Bratislava, Slovak Republic.
4. Institut de Chimie de Clermont-Ferrand, Université Clermont Auvergne, CNRS, F-63000 Clermont–Ferrand, France.
5. Department of Environmental Engineering, School of Resources and Environmental Science, Wuhan University, 430079 Wuhan, PR China.

*Correspondence: [olivier.monfort@uniba.sk](mailto:olivier.monfort@uniba.sk); +421290142141

**Table S1** Physicochemical characteristics of tertiary effluents of WWTP Bratislava-Petrzalka collected on 10/10/2023 and provided by BVS a.s. (Bratislava Water Company).

| Test | Quantity |
| --- | --- |
| Chemical oxygen demand | 26 mg L^-1^ |
| Biological oxygen demand | 3 mg L^-1^ |
| Non-dissolved matter at 105 °C | 10 mg L^-1^ |
| Total inorganic nitrogen | 9.31 mg L^-1^ |
| Total phosphor | 0.55 mg L^-1^ |

**Table S2.** Comparison of different C-based adsorbents in the removal of sulfamethoxazole and related compounds.

| Adsorbent | Source | Activation or Modification | BET area (m^2^ g^−1^) | Pollutant | Efficiency  (mg/g) | Ref. |
| --- | --- | --- | --- | --- | --- | --- |
| CNT | Synthesized | Hydroxylation | 228 | Sulfamethoxazole | 0.697 | (Zhang et al., 2010) |
| Activated carbon | Purchased | Fe^3+^ Modification | 70.96 | Sulfamethazine | 3.04 | (Liu et al., 2017) |
| Biochar | Peanut shell biochar | Fe and Mn modification | 192.83 | Sulfamethoxazole | 16.56 | (Z. Li et al., 2023) |
| Hydrochar | Orange peels | H_2_O_2_ | 74.6 | Sulfamethoxazole | 1.971 | This work |

Li, Z., Tian, W., Chu, M., Zou, M., & Zhao, J. (2023). Molecular imprinting functionalization of magnetic biochar to adsorb sulfamethoxazole: Mechanism, regeneration and targeted adsorption. *Process Safety and Environmental Protection*, *171*, 238–249. https://doi.org/10.1016/J.PSEP.2023.01.024

Liu, Y., Liu, X., Dong, W., Zhang, L., Kong, Q., & Wang, W. (2017). Efficient Adsorption of Sulfamethazine onto Modified Activated Carbon: A Plausible Adsorption Mechanism. *Scientific Reports*, *7*(1), 1–12. https://doi.org/10.1038/S41598-017-12805-6

Zhang, D., Pan, B., Zhang, H., Ning, P., & Xing, B. (2010). Contribution of different sulfamethoxazole species to their overall adsorption on functionalized carbon nanotubes. *Environmental Science and Technology*, *44*(10), 3806–3811. https://doi.org/10.1021/ES903851Q/SUPPL_FILE/ES903851Q_SI_001.PDF


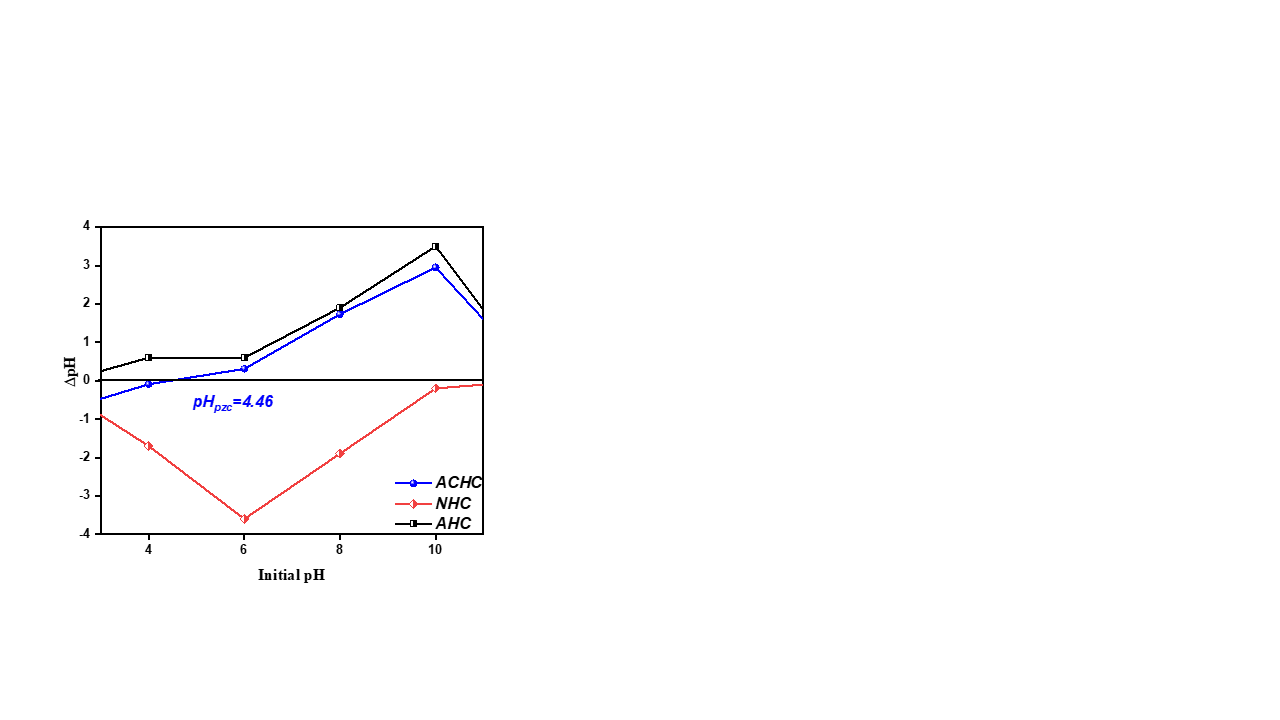


**Fig. S1.** pH drift method for HC, ACHC, AHC and NHC samples between pH 3-11.


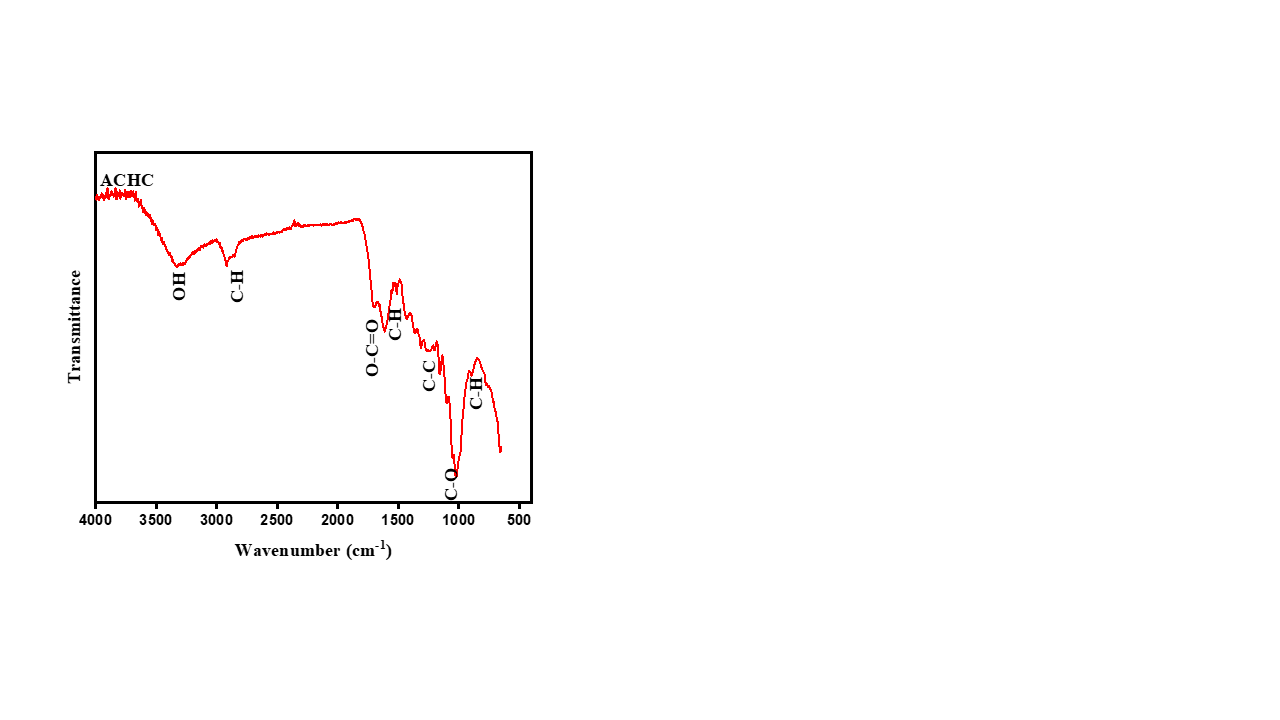


**Fig. S2.** FTIR of used hydrochars adsorbents


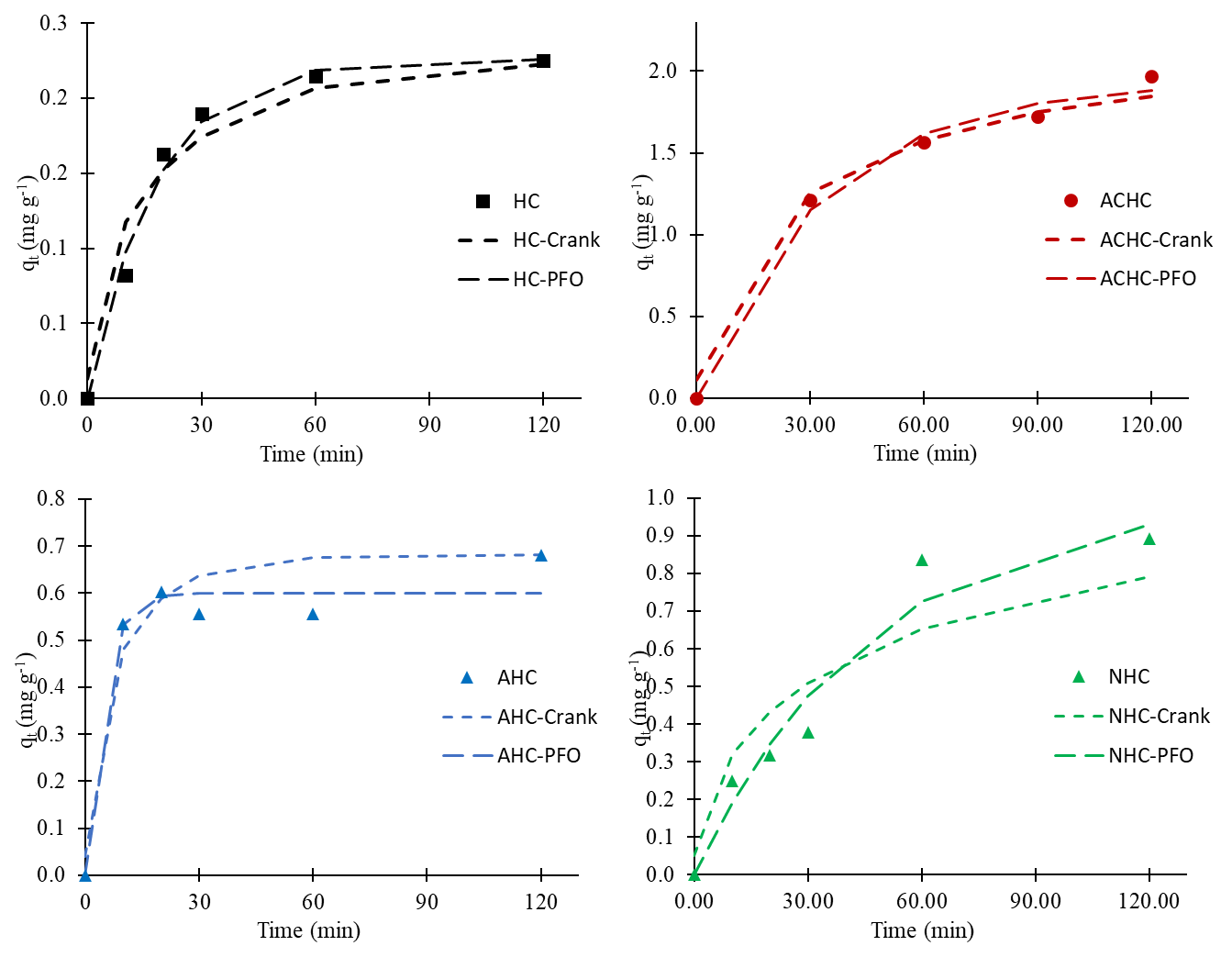


**Fig. S3.** Experimental adsorption isotherms (points) of HC, ACHC, AHC, and NHC samples fitted by Crank’s intraparticle diffusion model (Crank) and pseudo-first order model (PFO). Model parameters summarize Table 4.
